# Dual-Organ Transcriptomic Analysis of Rainbow Trout Infected With *Ichthyophthirius multifiliis* Through Co-Expression and Machine Learning

**DOI:** 10.3389/fimmu.2021.677730

**Published:** 2021-07-08

**Authors:** HyeongJin Roh, Nameun Kim, Yoonhang Lee, Jiyeon Park, Bo Seong Kim, Mu Kun Lee, Chan-Il Park, Do-Hyung Kim

**Affiliations:** ^1^ Department of Aquatic Life Medicine, College of Fisheries Science, Pukyong National University, Busan, South Korea; ^2^ Aquatic Disease Control Division, National Institute of Fisheries Science (NIFS), Busan, South Korea; ^3^ Korean Aquatic Organism Disease Inspector Association, Busan, South Korea; ^4^ Department of Marine Biology & Aquaculture, College of Marine Science, Gyeongsang National University, Tongyeong, South Korea

**Keywords:** dual-organ RNA-seq, *Ichthyophthirius multifiliis*, white spot disease, weighted gene co-expression network analysis, machine learning

## Abstract

*Ichthyophthirius multifiliis* is a major pathogen that causes a high mortality rate in trout farms. However, systemic responses to the pathogen and its interactions with multiple organs during the course of infection have not been well described. In this study, dual-organ transcriptomic responses in the liver and head kidney and hemato-serological indexes were profiled under *I. multifiliis* infection and recovery to investigate systemic immuno-physiological characteristics. Several strategies for massive transcriptomic interpretation, such as differentially expressed genes (DEGs), Poisson linear discriminant (PLDA), and weighted gene co-expression network analysis (WGCNA) models were used to investigate the featured genes/pathways while minimizing the disadvantages of individual methods. During the course of infection, 6,097 and 2,931 DEGs were identified in the head kidney and liver, respectively. Markers of protein processing in the endoplasmic reticulum, oxidative phosphorylation, and the proteasome were highly expressed. Likewise, simultaneous ferroptosis and cellular reconstruction was observed, which is strongly linked to multiple organ dysfunction. In contrast, pathways relevant to cellular replication were up-regulated in only the head kidney, while endocytosis- and phagosome-related pathways were notably expressed in the liver. Moreover, interestingly, most immune-relevant pathways (e.g., leukocyte trans-endothelial migration, Fc gamma R-mediated phagocytosis) were highly activated in the liver, but the same pathways in the head kidney were down-regulated. These conflicting results from different organs suggest that interpretation of co-expression among organs is crucial for profiling of systemic responses during infection. The dual-organ transcriptomics approaches presented in this study will greatly contribute to our understanding of multi-organ interactions under *I. multifiliis* infection from a broader perspective.

## Introduction

White spot disease caused by *Ichthyophthirius multifiliis* is a well-known condition that causes tremendous economic losses of trout farms but also other species of production fish (1–4). *I. multifiliis* can invade host epithelial layers, settle above the basal lamina, and cause tremendous damage to trout farms (5–7). Many studies have reported significant changes in innate (e.g., C3, macrophage activation with cytokine expression, serum amyloid A; SAA) and adaptive immune responses (e.g., IgT, IgM, and T and B lymphocytes) subsequent to *I. multifiliis* infection, but these findings are just the tip of the iceberg of immuno-physiological changes (8–15). In recent years, due to the development of advanced sequencing technologies, global transcriptomic analysis has become a robust tool for the study of immuno-physiological characteristics (16, 17). In the context of these trends, Syahputra et al. (4) monitored whole transcriptomic responses in the trout gill after *I. multifiliis* infection in an attempt to identify immunological mechanisms of response to infection. It is evident that the skin and gills are the primary *I. multifiliis* infection sites, but internal systemic responses in other immunologically significant organs (e.g., head kidney and liver) are also important (10, 11, 18). For instance, some studies (12, 19) found that several immune-related genes including complement factor, immunoglobulin, cytokine, and acute phase proteins were significantly differed depending on the type of organs (e.g., liver, head-kidney, and spleen). These results emphasize the importance of understanding global multi-organ transcriptomics, but there is currently not enough data on multi-organ transcriptomics responses under *I. multifiliis* infection in the rainbow trout.

Many studies have focused on methods of interpreting RNA-seq results, such as differentially expressed gene (DEG) analysis, which is able to identify significant alterations in gene expression (20). However, this method is of limited applicability to multi-organ RNA-seq results because DEG analysis is restricted to comparisons between only two groups. Also, an enormous number of DEGs can be retrieved from a dataset based on a given threshold value; accordantly, interpreting the importance of featured genes and their biological relevance can be very difficult (21–23). To overcome such problems, several statistical algorithms based on machine learning and weighted gene co-expression network analysis (WGCNA) are attracting a great deal of attention for their utility in interpreting massive amounts of transcriptomics data. These methods have advantages in efficiently analyzing multi-dimensional and/or multi-organ RNA-seq data and providing novel insights into systemic transcriptomic changes (23–26). However, a multi-organ transcriptomic atlas of any given hematopoietic organ has not yet been established. Here, we use dual-organ RNA sequencing (RNA-seq) approaches to characterize the despotic temporal transcriptome in detail during hematopoietic stem and progenitor cell expansion in the rainbow trout. Tools based on machine learning technology and WGCNA can make high-dimensional data analysis simple and help to identify featured genes, hub genes, and/or key factors (23, 27). If multi-dimensional transcriptomics results are interpreted through several well-known methods, the advantages of all methods can be maximized by compensating for the limitations of each. For instance, Deng et al. (28) combined the DEG and WGCNA methods to make up for the drawbacks of DEGs, which greatly contributed the understanding of the transcriptomic expression in association with the different pathological stages of renal cell carcinoma. Hence, WGCNA would help in understanding the transcriptomic changes in accordance with the *I. multifiliis* infection stages.

Therefore, the purposes of this study were to: 1) catalogue organ-enriched, organ-specific, and common gene expression profiles in the head kidney and liver, 2) investigate the interactions and relationship between these two organs, and 3) ultimately understand the systemic immuno-physiological changes in rainbow trout under *I. multifiliis* infection by means of serological and multi-organ transcriptomics data.

## Materials and Methods

### Fish and Samples

Severe *Ichthyophthirius multifiliis* infection occurred in a rainbow trout farm in Gangwon province, Korea with tens of thousands of juvenile rainbow trout (average = 61.7 ± 27.4 g). At the time, the water temperature was approximately 15°C. One week after the first death occurred, a 10% cumulative mortality rate was observed in cases of severe *I. multifiliis* infection in the gills and skin. At this time, 15 trout were randomly anesthetized with an excess of anesthetic agent (MS-222, Sigma) (Ich group). The trophont number was counted in the 1/2 gill arch to determine infection intensity, and all trout in the Ich group were found to be infected by *I. multifiliis*. Two weeks post-sampling, eight surviving trout with confirmed primary infection and recovery were additionally sampled as the recovery group (R-Ich group). Blood samples were collected from the caudal vein. Likewise, six trout of similar size without a history of *I. multifiliis* infection were sampled as the control (Con) group. Hematocrit (Ht) and serological factors (GOT, GPT, ALP, GLU, TCHO, TP, BUN, LDH, and Ca) were measured using a dry chemistry analyzer (FUJI DRI-CHEM 3000). Serum electrolyte levels (Na^+^, K^+^, and Cl^−^) were additionally checked using Fuji Dri-chem Na-K-Cl film; the reference fluid contained 100 mmol of sodium chloride (NaCl), 30 mmol of sodium bicarbonate (NaHCO_3_), 4 mmol of potassium dihydrogenphosphate (KH_2_PO_4_), and 0.1% NaN_3_. Based on the serological results, the osmolality of the serum was calculated using the following formula (29). The head kidney and liver were then collected and stored in RNAlater solution at −80°C.

$$\text{Calculated osmolality}{\text{L}}^{-1}=2\text{N}{\text{a}}^{+}(\text{mmol}\;{\text{L}}^{-1})+(\text{glucose}\;(\text{mg}\;\text{d}{\text{l}}^{-1})/18)+(\text{Urea}(\mathrm{mmol}\;{\text{L}}^{-1})/2.8)$$

### RNA Extraction and RNA-Seq Analysis

Three rainbow trout in each group were randomly selected for extraction of RNAs from the head kidney and liver using TRIzol reagent (Invitrogen, USA) following the manufacturer’s guideline. The concentration of RNA was quantified by Agilent 2100 BioAnalyzer and Nanodrop, and the sample with higher than 6 RNA integrity number RIN was used in this study. Libraries were prepared for 100 bp paired-end sequencing using a TruSeq Stranded mRNA Sample Preparation Kit (Illumina, CA, USA). Briefly, mRNAs were purified and fragmented from 1 μg of total RNA using oligo dT magnetic beads. These fragmented mRNAs were synthesized as single-stranded RNAs. Double-stranded cDNAs were then prepared. After the sequential end repair process, A-tailing, and adaptor ligation, the cDNA libraries were evaluated with an Agilent 2100 BioAnalyzer (Agilent, CA, USA). cDNAs were quantified with a KAPA library quantification kit (Kapa Biosystems, MA, USA) according to the manufacturer’s library quantification protocol. Following cluster amplification of denatured templates, paired-end (2 × 100 bp) sequencing was performed using an Illumina HiSeq 2500 sequencer (Illumina, CA, USA).

### Mapping, Assembly, and Selection of Differentially Expressed Genes

Low-quality reads were filtered according to the following criteria: reads containing more than 10% skipped bases (marked as “N”s), reads containing more than 40% bases with quality scores of less than 20, and reads with average quality scores of less than 20. The whole filtering process was performed using in-house scripts. Filtered reads were mapped onto a trout reference genome (30) using an aligner TopHat (31). Gene expression levels were measured with Cufflinks v2.1.1 (32) using the SwissProt gene annotation database. To improve measurement accuracy, the multi-read-correction and frag-bias-correct options were applied. All other options were set to default values. Differential expression analysis was performed using Cuffdiff (33).

### Pathway Analysis Using DEGs

Selected DEGs were functionally annotated using the KEGG database (34). A Z-score based on the number of increases or decreases in ko_id for each pathway was calculated using the following formula.

$$\text{Z}-\text{score}=\frac{\text{Number of up regulated}\;\text{k}{\text{o}}_{\text{id}}-\;\mathrm{Number of down regulated}\;\text{k}{\text{o}}_{\text{id}}}{\sqrt{\text{Number of ko}\_\text{id}}}$$

Based on the expression tendency of Z-score in the head kidney and liver, the pathways that were up- and down-regulated under *I. multifiliis* infection were classified. The pathways with the same number of up- and down-regulated DEGs as the Con and Ich groups were considered the etc. group. To investigate co-expression between organs, the pathways that showed up-regulation in both organs (PO-PO), up-regulation in the head kidney but down-regulation in the liver (PO-NE), down-regulation in the head kidney but up-regulation in the liver (NE-PO), and down-regulation in both organs (NE-NE) during *I. multifiliis* infection or recovery were categorized and visualized using Cytoscape (Ver. 3.6.1) (22, 35).

### Profiling of Featured Genes in Both Organs Using a Machine Learning Interface

To excavate the featured genes that differed between the control and Ich groups in the head kidney and liver RNA-seq analysis, Poisson linear discriminant analysis (PLDA) based on a machine learning method was implemented regardless of the organ. Briefly, twelve RNA-seq results from the control and Ich groups from both head kidney and liver were randomly split into eight samples (four samples from the control group and four from the Ich group) for the training set, and the remaining samples were used as the test set. The FPKM count in all transcripts was normalized by the “deseq median ratio normalization method”, calculated by dividing each FPKM sample by the geometric mean of the FPKM (36). The transformed PLDA model was generated using the training set, and featured genes were selected that effectively discriminated between the control and Ich groups. All statistical calculations and machine learning algorithms were performed using the MLSeq and DESeq2 packages in R (Ver. 3.6.2.) (23). Featured genes were annotated by Blast2GO software based on the GO database, KEGG, and SwissProt databases (34, 37). Featured genes were clustered based on GO and KEGG pathway, and visualized by Cytoscape (Ver 3.6.1.) (35).

### Weighted Gene Correlation Network Analysis (WGCNA)

The co-expression analysis of the FPKM counts of all 46,585 genes in 18 samples and the degree of infection (number of *I. multifiliis* microbes in the gill) were investigated using the WGCNA package in R (38). The similarity of the signed network was calculated using the formula below, as suggested by Langfelder and Horvath (38) and Kim et al. (26). In brief, blockwise modules were created under the following conditions: topological overlap matrix type = “Signed”, merge cut height = 0.25, verbose = 5, and soft thresholding power (β) = 16; the minimum and maximum number of genes in each module was 200 and 2,000, respectively. Also, gene significance (GS_i_) was calculated using the formula │cor (X_i_, degree of *I. multifiliis* infection)│, and modules with a P-value lower than 0.05 were selected as featured modules in accordance with *I. multifiliis* infection regardless of target organ. All genes in the featured modules were classified by gene ontology (GO) pathway, and functionally grouped GO assignments were made using ClueGO (39). The functional networks for pathways with P-value < 0.05 were drawn with a medium level of network specificity (0.55) using ClueGO and Cytoscape (35, 39).

$$\text{Similarity of signed coexpression}=\frac{1+\text{Cor}(\text{Xi},\text{Xj})}{2}$$

### Real-Time PCR

To validate sequence quantification, real-time PCR was performed targeting some selected DEGs (SAA, Hap, HSP70, Hmx, CD99, IgT, Wap65-1, and Col). cDNA synthesis and qPCR were performed following a previous study (40). Briefly, cDNAs were synthesized from 1 µg total RNAs. The qPCR mixture contained qPCR Green 2X Mastermix (Mbiotech, Korea) or qPCR probe 2X Mastermix (Mbiotech, Korea) and 400–500 nM of each primer (with 200–250 nM of supplementary probe added if necessary) for a total volume of 25 µl. Detail information on the primer, probe, and incubation conditions is shown in **Table S1**.

### Statistical Analysis

All hemato-serological results and osmolality are presented as average ± standard deviation. The differences between the control and *I. multifiliis* infection and/or recovery groups were tested for statistical significance using Student’s t-test or one way-ANOVA based on Duncan’s analysis. A P-value of less than 0.05 was taken to indicate a significant difference.

## Results

### Clinical Signs and Hemato-Serology

On average, 61.7 ± 27.4 ciliates per fish were found in the gills of trout in the *I. multifiliis* group (Ich). Gill aneurysms and hyperplasia were observed in the gills of all fish in the Ich group (data not shown). However, fish in the control (Con) and *I. multifiliis* recovery group (R-Ich) showed no clinical signs of *I. multifiliis* infection. The measurements of hematocrit (Ht), serological factors (Glutamic oxaloacetic transaminase, GOT; Glutamic pyruvic transaminase, GPT; Alkaline phosphate, ALP; Blood urea nitrogen, BUN; Glucose, GLU; Total cholesterol, TCHO; Total protein, TP; Lactate dehydrogenase, LDH; and Calcium, Ca) and electrolytes (Na^+^, Cl^−^, K^+^) in the Con, Ich, and R-Ich groups are shown in **Table 1**. The Ht readings of fish in the Ich and R-Ich groups were 21 ± 7 and 25 ± 5%, respectively, which are significantly lower than that of the control fish (39 ± 4%). Likewise, some serological indices, such as ALP, TCHO, and TP, were significantly down-regulated in both the Ich and R-Ich groups, but BUN and LDH levels were elevated only in the Ich group. GOT and GPT levels were significantly up-regulated only in the R-Ich group. Although Na^+^ concentration was constant in all groups in this study, K^+^ and Cl^−^ levels significantly differed in the Ich group (**Table 1**). In particular, the K^+^ concentration in the serum of fish in the Ich group was significantly higher than that of fish in the control group.

**Table 1:** Hematological and serological changes among groups (Control, Con; *I. multifillis* infected group, Ich; and *I. multifiliis* recovery group, R-Ich).

	Group		
	Con	Ich	R-Ich
Hematocrit (%)	39 ± 4^a^	21 ± 7^b^	25 ± 5^b^
GOT (U L^−1^)	421.8 ± 167.8^a^	601.4 ± 201.1^a^	1623.9 ± 1227.8^b^
GPT (U L^−1^)	24.2 ± 11.3^a^	27.1 ± 7.9^a^	63.8 ± 65.1^b^
ALP (U L^−1^)	430 ± 197^a^	121.4 ± 157.4^b^	152.8 ± 30.7^b^
BUN (mg dl^−1^)	2.3 ± 0.5^a^	3.1 ± 0.7^b^	2.1 ± 0.3^a^
GLU (mg dl^−1^)	101.7 ± 6.6	83.1 ± 32.2	77.1 ± 17.2
TCHO (mg dl^−1^)	318.7 ± 91.1^a^	157.3 ± 78.4^b^	192.6 ± 54.2^b^
TP (g dl^−1^)	4.3 ± 0.6^a^	3.8 ± 0.5^b^	3.8 ± 0.5^b^
LDH (U L^−1^)	1426.7 ± 354.1^a^	3116.3 ± 1790.1^b^	1328.5 ± 607.8^a^
Ca (mg dl ^−1^)	12.5 ± 0.3	11.7 ± 1.2	12.0 ± 0.7
Na^+^ (mEq L ^−1^)	158.8 ± 8.0	152.5 ± 11.8	152.0 ± 3.0
K^+^ (mEq L ^−1^)	<1.0^a^	9.1 ± 2.3^c^	3.8 ± 1.8^b^
Cl^-^ (mEq L ^−1^)	103.5 ± 13.6^a^	88.8 ± 9.8^b^	95.5 ± 2.8^ab^

Different letters indicated statistically significant differences by Duncan’s multiple range test (P < 0.05).

### Sequencing and Genome-Guided Assembly

**Figure 1:**
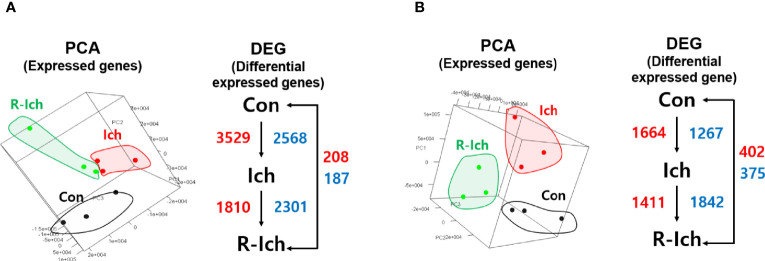
Principal component analysis (PCA) in the head kidney **(A)** and liver **(B)**. The red and blue numbers indicate up- and down-regulated DEG, respectively, for different comparison sets.

Eighteen cDNA libraries (nine head kidney and liver samples) were individually constructed and sequenced using the Illumina HiSeq platform. A total of 889,869,076 raw reads (453,045,574 and 436,823,502 raw reads for the head kidney and liver, respectively) were obtained. On average, 49,437,171 reads were produced for each sample. Of these reads, the majority (97.1%) passed quality control. Finally, 439,458,201 and 424,700,679 clean reads of the head kidney and liver, respectively, were mapped onto the reference genome. Of those, 356,208,296 and 337,667,476 reads were successfully mapped, respectively (average mapping rate = 80.31%) (**Table S2**). Among 41,978 predicted genes in the rainbow trout, the average numbers of expressed genes in the Con, Ich, and R-Ich groups were 34,061, 31,854, and 34,660, respectively, in the head kidney and 29,875, 28,899, and 30,467 in the liver. Based on the fragments per kilobase per million mapped reads (FPKM) of expressed genes, 3D-PCA plots were prepared for the head kidney and liver, as shown in **Figures 1A, B**, respectively.

### Selection of DEGs

Genes with a q-value of less than 0.05 were selected as DEGs (41). Six DEG sets, including comparisons of the Con *vs.* Ich, Con *vs.* R-Ich, and Ich *vs.* R-Ich groups in the head kidney and liver, were calculated. For the head kidney, 6,097 DEGs (up-regulation: 3,529; down-regulation: 2,568), 395 DEGs (up-regulation: 208; down-regulation: 187), and 4,111 DEGs (up-regulation: 1,810; down-regulation: 2,301) were found in the comparison of Con *vs.* Ich, Con *vs.* R-Ich, and Ich *vs.* R-Ich, respectively. Likewise, 2,931, 777, and 3,253 genes in Con *vs.* Ich, Con *vs.* R-Ich, and Ich *vs.* R-Ich were identified as DEGs in the liver (**Figure 1**).

### Pathway Analysis During *I. multifiliis* Infection and Recovery in a Single Organ

**Figure 2:**
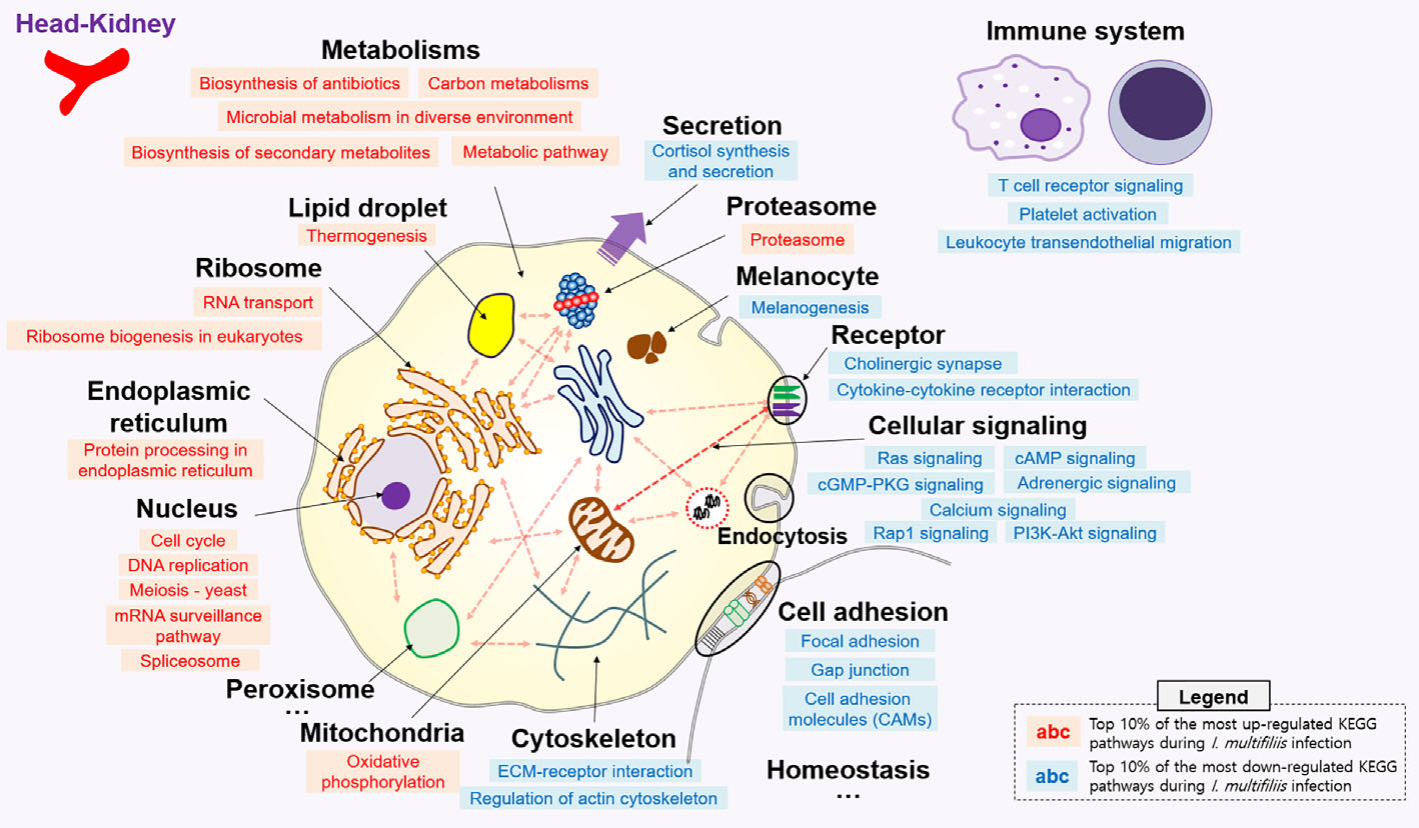
Top 10% enriched KEGG pathways comprised of 16 up- and 19 down-regulated pathways in the head-kidney of rainbow trout during *I. multifiliis* infection. The red and blue letters indicate up- and down-regulated KEGG pathways, respectively, during *I. multifiliis* infection. More detailed information is available in **Table S4**.

**Figure 3:**
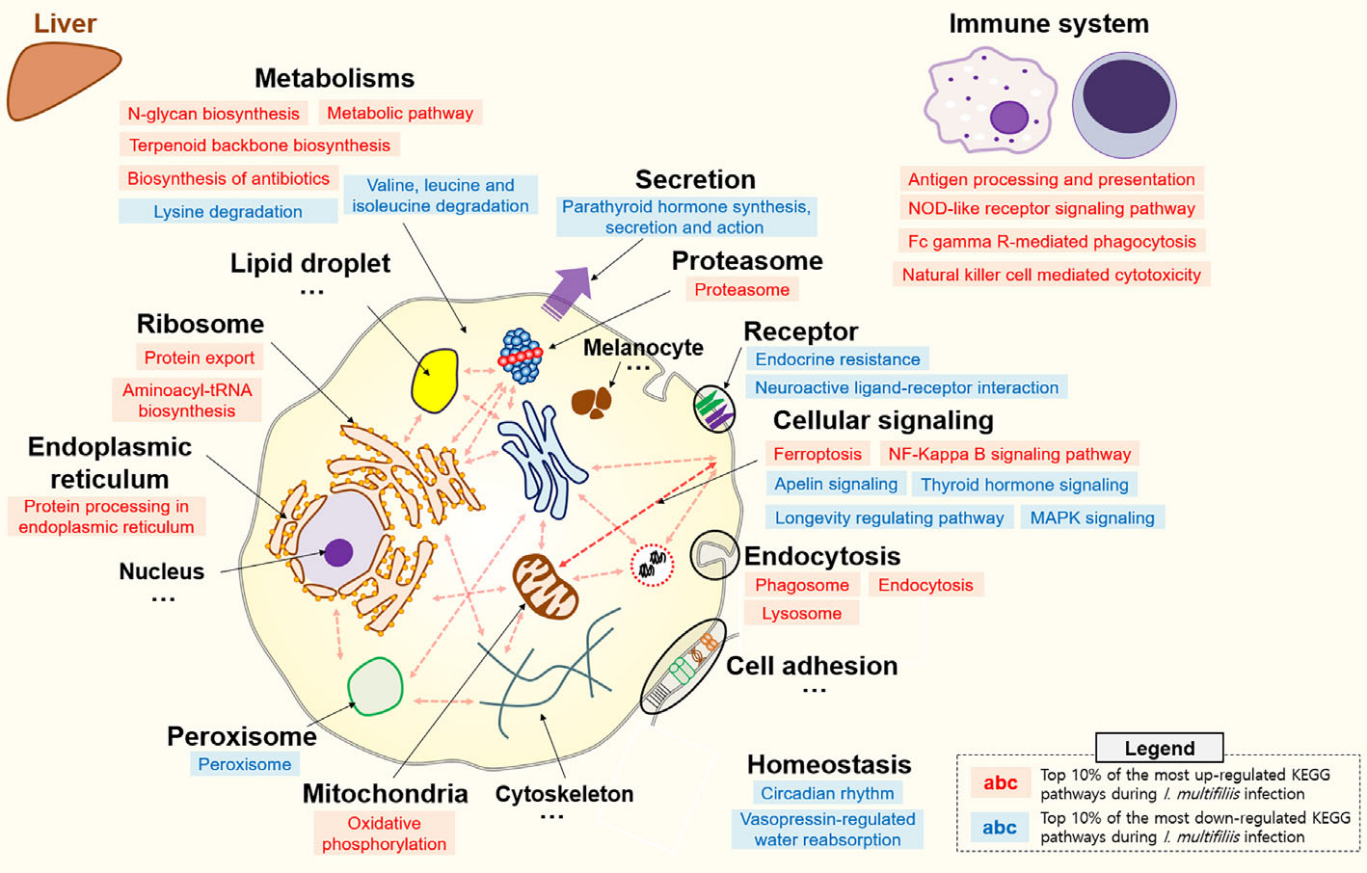
Top 10% enriched KEGG pathways comprised of 18 up- and 12 down-regulated pathways in the liver of rainbow trout during *I. multifiliis* infection. The red and blue letters indicate up- and down-regulated KEGG pathways, respectively, during *I. multifiliis* infection. More detailed information is available in **Table S5**.

All KEGG-annotated DEGs were categorized in hundreds of KEGG pathways. At least one DEG was involved in 367 and 356 different KEGG pathways in the head kidney and liver, respectively. Genes with Z-scores in the positive direction (Z-score > 0) and negative direction (Z-score < 0) in the comparison of the Con *vs.* Ich groups were considered to be up- and down-regulated KEGG pathways during *I. multifiliis* infection. Overall, the number of down-regulated pathways (51%; 182/357) was greater than that of up-regulated pathways (46%; 163/357) among pathways with at least one DEG in the head kidney during *I. multifiliis* infection. However, the liver showed opposite results: 51% (183/356) and 40% (144/356) up- and down-regulated pathways, respectively, among expressed KEGG pathways (**Table S3**). The top 10% most profoundly altered pathways were selected by calculating the sum of |Z-score| in Con *vs.* Ich after excluding human disease-relevant pathways. This produced a total of 16 and 18 featured up- and down-regulated pathways, respectively, in the head kidney (**Figure 2** and **Table S4**). Metabolism (ko01100), RNA transport (ko03013), and the spliceosome (ko03040) were the first, second, and third most highly up-regulated pathways, respectively, while focal adhesion (ko04510), Rap1 signaling (ko04015), and ECM-receptor interactions (ko04512) were the most down-regulated pathways in the head kidney (**Table S4**). Likewise, 18 and 12 pathways were selected as the top 10% up- and down-regulated pathways, respectively, in the liver during *I. multifiliis* infection (**Figure 3** and **Table S5**). The proteasome (ko03050), protein processing in the endoplasmic reticulum (ko04141), and the lysozyme (ko04142) were the top three up-regulated pathways, while endocrine resistance (ko01522), MAPK signaling (ko04013), and circadian rhythm (ko04710) were the three most down-regulated pathways in the liver (**Table S5**).

### Co-Expression Analysis With Transcriptomic Responses in the Head Kidney and Liver Under *I. multifiliis* Infection and Recovery

**Figure 4:**
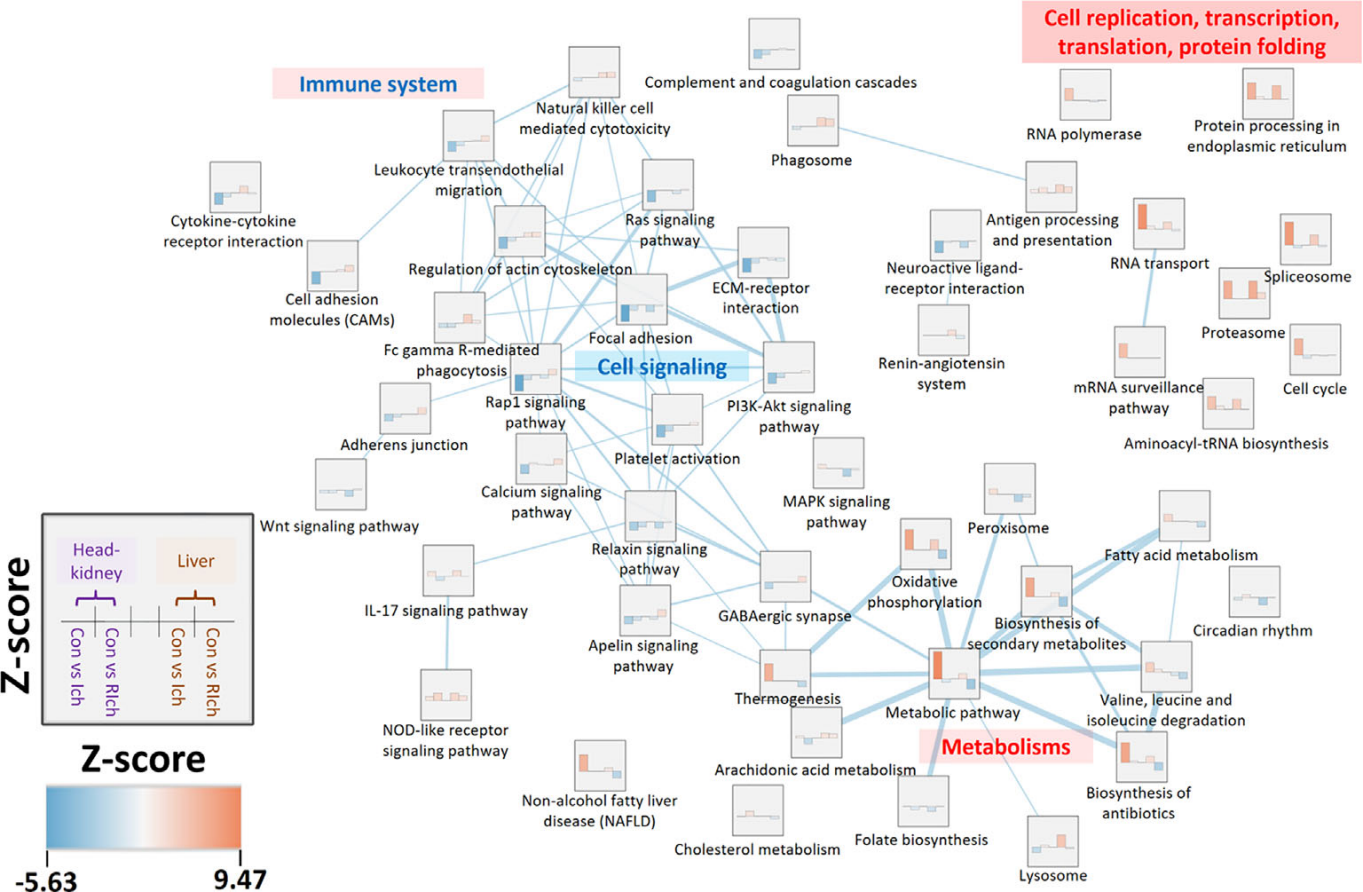
Enrichment network analysis based on top 10% co-expressed pathways in the head kidney and liver during *I. multifiliis* infection and recovery. Z-scores range from blue (lower Z-score) to red (higher Z-score). Edges and edge thickness, which represent biological similarity and the degree of overlap between two pathways, are shown in the light blue line. More detailed information is available in **Tables S6, S7**.

In order to investigate the shared and independent DEGs and pathways between organs during *I. multifiliis* infection and recovery, the co-expressed pathways and DEGs were profiled. For the Ich and R-Ich groups, pathways with positive Z-scores in both the head kidney and liver were classified as PO-PO, while pathways with negative Z-scores in both organs were considered NE-NE. Likewise, pathways with a negative Z-score in the head kidney but a positive Z-score in the liver were classified as NE-PO, and pathways where Z-score > 0 and Z-score < 0 in the head kidney and liver, respectively, were called PO-NE (**Figure 4** and **Tables S6, S7**). During *I. multifiliis* infection, the metabolic (ko01100) and proteasome (ko03050) pathways were most highly represented in the PO-PO direction. On the other hand, the focal adhesion (ko04510) and circadian rhythm (ko04710) pathways had the lowest Z-scores in the head kidney and liver, and the featured pathways in the NE-PO and PO-NE directions were cell adhesion molecules (CAMs) (ko04514) and lysosome (ko04142), and cell cycle (ko04110) and MAPK signaling pathway (ko04013), respectively (**Tables S6, S7**). Approximately 28, 25, 17, and 10% of pathways in the Con *vs.* Ich group were included in the PO-PO, NE-NE, NE-PO, and PO-NE directions, while 14, 7, 23, and 8% of pathways in the Con *vs.* R-Ich group belonged to the PO-PO, NE-NE, NE-PO, and PO-NE directions (**Table S8**). The Z-score and the similarity between groups in the top 10% featured co-expressed pathways were visualized using an enrichment-network map (**Figure 4**). In general, pathways related to cell replication, transcription, and cell cycles were highly expressed in the head-kidney, while protein folding and metabolism were mutually activated in both organs. But, the cellular signaling pathways such as focal adhesion, ECM-receptor interaction, and Rap1 signaling pathway were down-regulated in the head kidney. Noticeably, only the head kidney, not the liver, showed overall down-regulation of the immune system in the top 10% pathways (**Figure 4**). In the figures, the dotted line connecting the DEGs and relevant pathways, along with the red and blue colors in nodes and edges, indicate up- and down-regulated DEGs in the head kidney and liver (**Figures S1–S4**). Only a few DEGs were shared among organs, and most DEGs belonged only to either the head kidney or liver.

### Featured Genes and Network Analysis Based on PLDA Analysis

**Figure 5:**
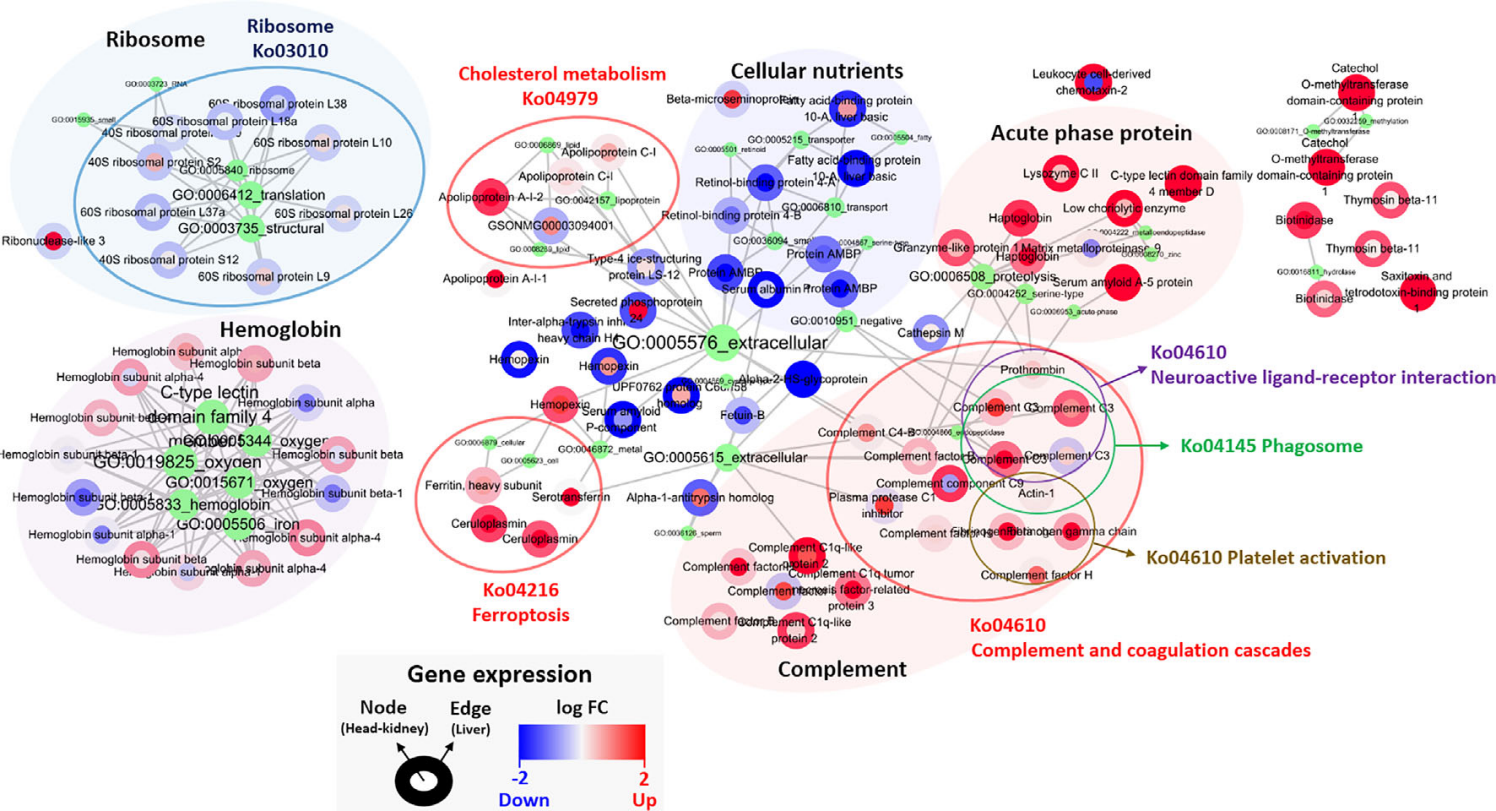
Classified genes from PLDA calculation. Nodes and edges in the circles color are the areas for the gene expression in the head-kidney and liver, respectively, and red and blue color show up- and down-regulated genes. The green circle represents GO pathway, and its size and letters indicate the number of genes belonging to each GO pathway. The gene(s) belonging to GO pathway(s) is marked by gray lines. Each gene expression is described in **Table S9**.

One hundred featured genes among the total of 46,585 genes were selected after applying the Poisson linear discriminant (PLDA) algorithm, and all the featured genes were standardized by the gene expression level of the control group (**Table S9**). The fold changes in the head kidney and liver were indicated in the node and edge parts of each featured gene, and network analysis was implemented with clustering by shared Gene ontology (GO) and Kyoto Encyclopedia of Genes and Genomes (KEGG) pathways (**Figure 5**). The results showed that most featured genes were involved in cholesterol metabolism (Ko04979), ferroptosis (Ko04216), and the complement and coagulation cascades (Ko04610); also, the levels of several acute-phase proteins such as haptoglobin and serum amyloid A were increased. In addition, there was a reduction in the expression of genes associated with ribosomes and cellular nutrients (**Figure 5**).

### Featured Modules Based on WGCNA

**Figure 6:**
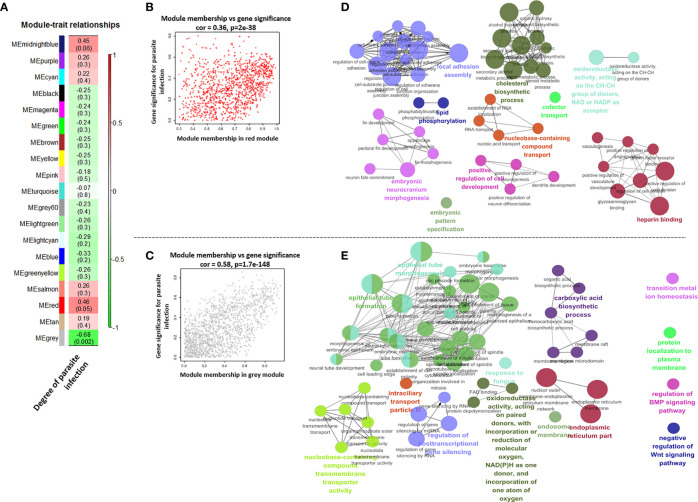
Correlations between each module’s eigen-gene (the first principal component among the genes in the module) and degree of parasite infection **(A)**. Scatter plots for module membership and gene significance in the red and gray module **(B, C)**. The size of circles and edge thickness represents the number of enriched genes in individual functional groups and the degree of overlap (biological similarity) between two pathways in the red and gray module, respectively **(D, E)**.

The module in WGCNA is defined as highly interconnected gene clusters, and a total of 19 modules were identified in the module classification, and the red and gray modules out of 19 modules were selected as featured modules (**Figures 6** and **S5**). The red module is highly positively correlated with the degree of *I. multifiliis* infection (correlation coefficient = 0.46, P-value = 0.05), while the gray module is strongly negatively correlated (correlation coefficient = −0.68, P-value = 0.002). In the red module, the focal adhesion assembly, lipid phosphorylation, embryonic neurocranium morphogenesis, embryotic pattern specification, positive regulation of cell development, nucleobase-containing compound transport cofactor transport, cholesterol biosynthesis, cofactor transport, oxidoreductase activity, and heparin-binding pathways were the major functional groups that were positively correlated with the degree of *I. multifiliis* infection. On the other hand, epithelial tube morphogenesis, epithelial tube formation, nucleobase-containing compound transmembrane transport activity, regulation of post-transcriptional gene silencing, intraciliary transport particle B, oxidoreductase activity, response to fungus, carboxylic acid biosynthesis, endosomal membrane, endoplasmic reticulum parts, transition metal ion homeostasis, protein localization to the plasma membrane, regulation of BMP signaling pathway, and negative regulation of Wnt signaling pathway were featured functional groups in the gray module, which were down-regulated in accordance with the number of parasites in the gills (**Figure 6**). Detailed information on each pathway, such as GO ID, term, ontology source, and P-value, is shown in **Table S10**.

### RNA-Seq Validation Using qPCR

**Figure 7:**
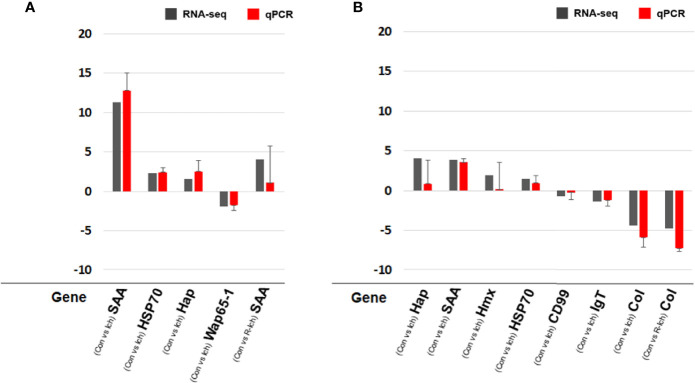
Comparison of gene expression between the RNA-seq and qPCR results in the liver **(A)** and head kidney **(B)** targeting SAA, Hap, HSP70, Hmx, CD99, IgT, Wap65-1, and Col. The qPCR results in the bar graph are shown as mean ± standard deviation.

In order to validate RNA-seq, eight DEGs were selected in consideration of the biomarkers known to be meaningful for the infectious disease and/or stress environment in former studies and the pattern of transcriptomic expression in this study (40, 42, 43). The expression levels of some DEGs were validated using qPCR. Relative fold changes compared to control were calculated with the ΔCt or ΔΔCt method using an internal control (Ef-1α). A total of eight genes (SAA; haptoglobin, Hap; heat shock protein, HSP70; hemopexin, Hmx; CD99 antigen-like protein, CD99; IgT; warm temperature acclimation protein, Wap65-1; collagen alpha-1 chain, Col) were selected to validate DEGs in the liver or head kidney for the Ich and R-Ich groups. Although the gene expression levels differed slightly between the qPCR and RNA-seq results, the direction and tendency were quite consistent. In the qPCR results, SAA and Col were the most up- and down-regulated gene; the same is true in the RNA-seq results (**Figure 7**).

## Discussion

Since fish gills and skin are the major portals of entry of *I. multifiliis*, RNA-seq analysis targeting the primary infection site was conducted accordantly in the former study (19). Syahputra et al. (4) investigated global transcriptomic expression in the gills of rainbow trout infected with *I. multifiliis*, and found the most significantly altered genes were related to immune pathways such as NOD-like receptor signaling, toll-like receptor signaling, platelet activation, chemokine signaling, leukocyte transendothelial migration, antigen processing and presentation, and T and B cell receptor signaling. However, not many studies have been done on transcriptomics analysis of the internal immunogenic organs despite the significant changes in genes related to the innate and adaptive immune system that occur following *I. multifiliis* infection (44). The gene expression of cytokines, chemokines, and complement factors in multiple organs (e.g., gill, spleen, liver, and head kidney) were compared using qPCR in previous studies (12, 19), and the authors showed notable different transcriptomic responses after an *I. multifiliis* infection. But, they profiled only a few selected genes out of more than tens of thousands, which are still insufficient to understand overall responses happening during the infection. This problem has been a major obstacle to understanding the systemic host responses against white spot diseases up until now. In this study, we successfully profiled major transcriptomics in the internal immunogenic organs, and the host biological interaction against *I. multifiliis* was investigated through the integration of the differential expression in major organs. Among immune-relevant organs, the fish head kidney and liver are well-known primary lymphoid tissues responsible for the immune system, central metabolism (carbohydrate, protein, and lipid metabolism) and detoxification (42, 45, 46). In particular, the liver is a central part of not only the primary immune system (responsible for phagocytosis and complement activation) but also the adaptive immune system (lymphocyte proliferation and antigen presentation) (47). Nevertheless, transcriptomic studies targeting these organs in the rainbow trout under *I. multifiliis* infection have not been well-described. Accordantly, we focused on profiling and interpreting dual-organ transcriptomic responses (head kidney and liver) to *I. multifiliis* infection through several types of multi-dimensional data analysis, such as DEGs, co-expression, and machine learning methods, and investigated the systemic interactions among the major organs while considering previous transcriptomic results in the gills (19). Although this study has limitations in that only three trout for each group were used and it was very difficult to obtain accurate disease history of the fish sampled from the fish farm, the results presented in this study greatly contribute to the systems biology of *I. multifiliis* infection in the fish.

During the course of *I. multifiliis* infection, some biological pathways were mutually expressed in the same direction in both the head kidney and liver, but other pathways were significantly altered in only one organ or had opposite expression patterns in the two organs. For example, pathways relevant to the endoplasmic reticulum (ER), mitochondria and proteasome, protein processing in the ER, oxidative phosphorylation, and proteasome were up-regulated in both organs. In general, the major function of the ER is to fold and process proteins (48). However, when many unfolded and misfolded proteins are present due to abnormal conditions and diseases, the ER increases its folding capacity to maintain homeostasis (49, 50). Increased expression of ER-related pathways may represent a host strategy to control the numerous inappropriate proteins caused by homeostasis imbalance and/or relevant indirect biological stress because of *I. multifiliis* infection as shown in some parasitic and bacterial infections (50, 51). Inácio et al. (52) demonstrated the protozoa infection (*Plasmodium berghei*) stimulated the ER-resident unfolded protein responses, which greatly contributed to parasite development by showing the decrease parasite load under knocking-down the ER transcription factor (cAMP responsive element-binding protein). This implied that the expression of ER-related pathways would be closely linked to the parasite infection in teleost as well. However, since *I. multifiliis* is an extracellular parasite unlike an intracellular parasite, *Plasmodium berghei*, further studies are needed to verify the direct relationship between ER and *I. multifiliis* infection.

In particular, when the system is overwhelmed by an excessive amount of inappropriate proteins, remaining abnormal proteins will be processed through the ER-associated protein degradation (ERAD) pathway and subsequently degraded by proteolytic activities in the proteasome (53). Hence, the high expression of the proteasomal genes seen in this study may be attributable to ERAD. This implies that rainbow trout under physiologically unstable conditions including *I. multifiliis* infection can increase expression of genes involved in the ER, mitochondria, proteasome, protein processing in the ER, and oxidative phosphorylation in different types of tissues. Also, genes linked to oxidative phosphorylation, the major metabolic pathway through which ATP is produced by cellular respiration, and the metabolism were strongly linked to significant responses to the infection in both organs (53, 54). Abdel-Hafez et al. (55) showed that liver glycogen can be depleted due to loss of osmoregulation during *I. multifiliis* infection, leading to a significant reduction in serum glucose levels, despite excessively high expression of metabolism-related and oxidative phosphorylation pathways. These transcriptomic responses with low glucose levels are the consequences of rapid energy depletion during *I. multifiliis* infection, and large expenditures of energy and metabolites could help to repair any damage and counteract the pathogen (56). In addition, most pathways related to cell replication, such as the cell cycle, DNA replication, meiosis, mRNA surveillance, and spliceosome pathways, were among the top 10% most up-regulated KEGG pathways in the head kidney, though they did not among the top 10% of pathways in the liver during *I. multifiliis* infection. Severe anemia, pale gills, and low numbers of erythrocytes are known as the major clinical signs of *I. multifiliis* infection (55, 57, 58). Likewise, we observed not only pale gills during infection but significantly lower Ht in the Ich and/or R-Ich groups, which implies that the host was in a state of considerable erythrocyte deficiency. Accordantly, infected trout should produce more erythrocytes than normal, and up-regulation of cell replication pathways provide evidence of activation of hematopoiesis in an attempt to counteract erythrocyte destruction.

Since the head kidney is the primary hematopoietic organ in the teleost, researchers generally think that high expression of cell replication- and hemoglobin-related genes occurs in the head kidney but not the liver (45, 46, 59). An increase in immunological pathways was a representative biological response that showed opposite directions of transcriptomic expression between the head kidney and liver. Overall, in infected trout, greater activation of immunological pathways in the liver was correlated with reduced activation of such pathways in the head kidney. Notably, major chemo-attractive and immune-relevant pathways were down-regulated in the head kidney, but slightly increased in the liver. Likewise, the expression of leukocyte cell-derived chemotaxis-2, one of the featured genes identified from PLDA analysis, was greatly increased in the liver but decreased in the head kidney, strongly indicating movement of leukocytes from the head kidney to the gills and other sites. Unlike other major bacterial and viral diseases that infect internal organs, the major *I. multifiliis* infection sites are the gills and skin, not the head kidney, and thus this difference is thought to cause leukocyte migration from hematopoietic tissues to the gills and skin (60). To allow for easy leukocyte migration to other tissues, the host needs to decrease the chemo-attractive and immune responses in the head kidney. Since platelet activation and T cell receptor signaling pathways are involved in the stimulation of cytokine expression and the mediation of leukocyte movement, down-regulation of these pathways in only the head kidney could help to maintain a low level of chemo-attractive responses, thereby releasing leukocytes from the head kidney (4, 61, 62). Syahputra et al. (4) also showed changes in the expression of pathways related to chemokine signaling and leukocyte activation, such as the Toll-like and NOD-like signaling pathways, in the gills, as in the liver in the current study. These patterns were also observed in the liver, which showed significant changes in the expression levels of genes related to major immunological responses such as antigen processing and presentation, NOD-like signaling, Fc gamma R-mediated phagocytosis, and natural killer cell-mediated cytotoxicity. Also, Jørgensen et al. (12) also observed overall up-regulation of cytokines (IL-1β, IL-6, INF-γ, and TNF-α) in the liver of rainbow trout at 1–4 week(s) post challenge with *I. multifiliis*, but no changes or down-regulation of INF- γ were found in the head-kidney of fish at the same sampling time point. Although the liver is not a primary infection site, Castro et al. (42) emphasized that it is an important immunocompetent organ in that numerous pathogens can be ingested orally and detected in the gastrointestinal tract *via* the portal vein. Since *I. multifiliis* have free living stages in the water, trout could accidentally uptake many live or dead *I. multifiliis* including their protein and DNA molecules. Also, the possibility that macrophages in the infection sites would move to the liver through the blood, and then present the *I. multifiliis* antigens to intraparenchymal macrophages should be considered (42). The activation of uptake of extracellular materials and molecules, including phagocytosis and endocytosis indicates that the liver has very important immunological roles against *I. multifiliis* infection. But, further studies are necessary to clarify the mode of action of activated phagocytosis and antigen processing and presentation in the liver under *I. multifiliis* infection. In the recovery stage, when compared to the Ich group, thousands of DEGs were found, but only several hundred DEGs (less than 10% of the number of DEGs between the Con and Ich groups) were found in the comparison between the Con and R-Ich groups. Also, most genes that were significantly altered during *I. multifiliis* infection returned to similar expression levels in the Con group. For serological results, however, the differences between the Con and Ich groups were much greater than between the Con and R-Ich groups, which is the opposite of the transcriptomic results. This would imply that although transcriptome responses were recovered in accordance with the recovery of *I. multifiliis* infection, the accumulated damages to the host in R-Ich group had not yet been recovered.

**Figure 8:**
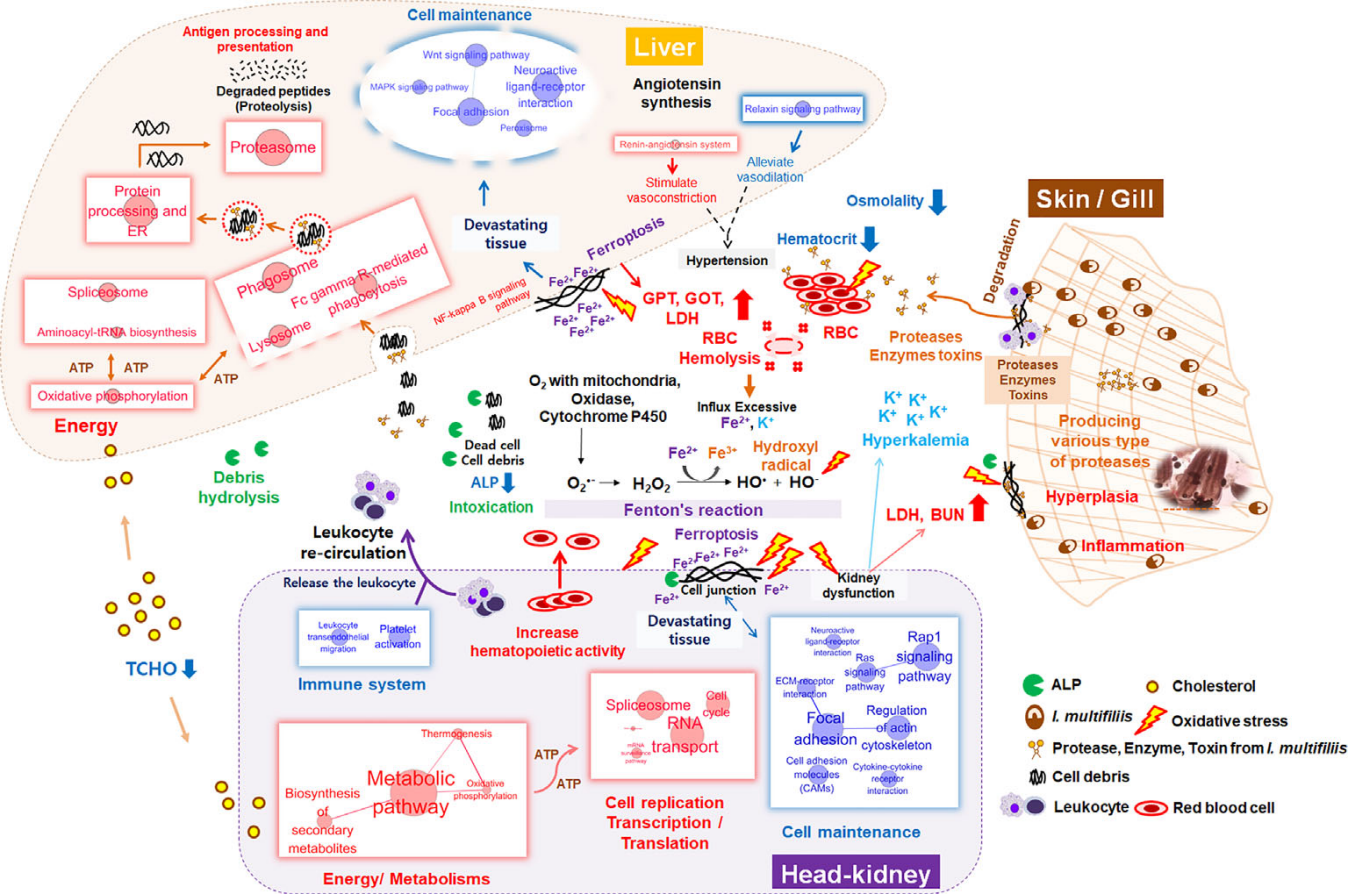
Schematic diagram of systemic changes in the liver, head kidney, blood, and primary infection sites (e.g., gill and skin) during *I. multifillis* infection. The size of red and blue circles with letters represents the number of enriched genes in each KEGG pathway, and edge thickness between two pathways indicates the degree of overlap (biological similarity) during *I. multifiliis* infection.

To invade the mucosal and epidermal layer, *I. multifiliis* can cause cellular destruction and histo-pathological changes such as necrosis, inflammation, and epidermal proliferation (7, 55, 63). Numerous proteases and lytic enzymes from parasites are well-known virulence factors that can destroy host tissues, such as the extracellular matrix and interstitial tissues, through catabolism of host membranes and cytoadherence (64). Several studies have reported on the existence of multiple types of cysteine proteases in *I. multifiliis* according to gelatin-precast zymography and genomic information from the macro-nucleus (7, 64–66). These virulence factors can directly affect erythrocytes, thus inducing hemolysis and anemia, as shown in this study (decrease in Ht from 39 to 21% after *I. multifiliis* infection) as well as other studies (55, 57, 58). When erythrocytes are ruptured, excessive Fe^2+^ and K^+^ flow into the blood. Although ferrous (Fe^2+^) is necessary for the function of NADPH oxidase, cytochrome P450, and the electron transport system, excessive Fe^2+^ catalyzes hydrogen peroxidase (H_2_O_2_) to hydroxyl radical (^•^OH) in “Fenton’s reaction”, the latter of which can induce greater oxidative stress than H_2_O_2_ (67, 68) (**Figure 8**). Hence, massive hemolysis caused by *I. multifiliis* infection can trigger an influx of Fe^2+^ in the serum and cause systemic DNA damage, lipid peroxidation, oxidative stress, and ferroptosis. Ferroptosis is an iron-dependent cell death mechanism first defined by Dixon et al. (69). Interestingly, we observed that cell death pathways, such as the ferroptosis and NF-Kappa B signaling pathways, had high Z-scores in both organs. In the context of dysfunction and destruction of both organs, pathways relevant to cellular homeostasis, such as the functional cytoskeleton (ECM-receptor interaction, regulation of actin cytoskeleton), cell adhesion (focal adhesion, gap junction, cell adhesion molecules), and cellular signaling (Ras signaling, cGMP-PKG signaling, cAMP signaling, adrenergic signaling, calcium signaling, Rap1 signaling, and PI3K-Akt signaling), were simultaneously down-regulated in the Ich group, indicating that the function of the kidney was compromised. Additionally, impairment of the kidney and liver was verified by serological evidence (GOT, GPT, and BUN), electrolytes (K^+^), and osmotic imbalances in the blood. Potassium is a major intracellular electrolyte. Approximately, 98% of K^+^ is located in intracellular fluid (ICF). Destruction of cells (e.g., cell lysis, hemolysis) can lead to severe hyperkalemia and result in broad physiological disorders (70). In general, excessive K^+^ in extracellular fluid (ECF) is processed by the kidney. However, compromised renal function such as renal failure can lead to low K^+^ excretion through urine and failure to recovery K^+^ in the serum (71, 72). Likewise, the observed increase in BUN, which is readily eliminated by normally functioning kidneys and gills, is another indication of kidney and gill dysfunction in the teleost (73–75). Additionally, the levels of GOT and GPT, which are well-known biomarkers of hepatocyte damage, were approximately three to four times higher in the R-Ich group than the Con group, which provides clear evidence of liver dysfunction and damage. Even though *I. multifiliis* is normally infected in the skin and gills, we found that its virulence mechanisms and toxins systemically influence the host. The gills and skin are the same in that they are the site of primary infection, but their transcript responses under *I. multifiliis* infection may be different because of biological function, species, and etc. (19, 76). However, even though *I. multifiliis* was not directly infected with internal organs (e.g., live, head-kidney), it could greatly influence on systemic damages in rainbow trout as shown in **Figure 8**.

One hundred featured genes were selected based on a machine learning method using the PLDA algorithm, which is a widely known nearest shrunken centroids algorithm for discrimination between different groups in micro-array and RNA-Seq data (77). This method is particularly useful for finding genes that are sensitive markers of the difference between control and treatment groups, and is specialized for the discovery of potential biomarkers or featured genes (23, 78). Also, we analyzed the transcriptomic results without considering the target organ to identify major systemic expression regardless of tissue type. Since this is a good approach to finding the key genes that are expressed, the pathways that are highly linked to key genes represent the major host responses to *I. multifiliis* infection in both organs. As a result, ferroptosis, complement and coagulation cascades, and cholesterol metabolism can be regarded as featured pathways that are co-expressed in multiple organs rather than confined to a single organ under *I. multifiliis* infection. Although some pathways (e.g., ferroptosis, phagosome) and genes were also investigated in the DEG analysis, others such as complement and coagulation cascades and hemoglobin relevant genes, not classified as DEGs, were more notably identified in PLDA. These results indicated that the application of different approaches for RNA-seq analysis, including machine learning, can broaden the perspectives to investigate biological meaningful pathways that could potentially be missed in a single method. In WGCNA, co-expression networks were generated using the expression tendency of all individual genes and the degree of infection, and red and gray modules were used to represent significant gene clusters that most positively and negatively correlated with parasite burden in this study. More severe *I. multifiliis* infection led to higher expression of the focal adhesion assembly, positive cell development regulation, and cholesterol biosynthesis pathways, which reflect the process of recovery. Also, heparin-binding growth factors including fibroblast growth factor and vascular endothelial growth factor in the red module are known to play roles in wound healing and vasculature development, which are important modes of action for recovery from an infection (79). In addition, the many pathways related to cell structures and signaling, such as the epithelial tube, endoplasmic reticulum, and plasma membrane, observed in the gray module can be interpreted as representing the cellular damage caused by *I. multifiliis* infection. This implies that the degree of *I. multifiliis* infection would mainly affect cellular signaling, development, and maintenance in both the head kidney and liver, indicating that the infection causes systemic responses, not only local responses in specific organ(s) as illustrated in **Figure 8**.

In this study, we profiled dual-organ transcriptomics during *I. multifiliis* infection and recovery through several strategies for multi-dimensional data interpretation. Most highly expressed biological pathways include protein processing in the ER, oxidative stress, and proteasome in both organs. However, the majority of physiological responses showed marked differences between organs in response to *I. multifiliis* infection. The pathways involved in cell production and movement were most highly affected only in the head kidney, while endocytosis and some crucial cellular signaling responses such as NF-kB signaling were more notably expressed in the liver. Moreover, major immune pathways in the head kidney, especially leukocyte signaling and trans-endothelial migration, as well as chemo-attractive pathways were primarily down-regulated, but strong activation of antigen processing and presentation, leukocyte-mediated cytotoxicity, and phagocytosis was observed in the liver. Significant changes in the levels of serological factors (GOT, GPT, BUN, and K^+^) along with lower Ht reflect the destruction of tissues and hemolysis. Massive amounts of K^+^ and Fe^2+^ were released to the serum by hemolysis. Free Fe^2+^ stimulates toxic oxidative stress (•OH) through Fenton’s reaction, which induces systemic ferroptosis and leads to multiple organ dysfunction syndromes. Following *I. multifiliis* infection, control of the expression of various immune systems, restoration of damaged tissues and organs, side effects, and responses to infection simultaneously occur inside the body. The dual-organ transcriptomic strategy used in this study provides a robust tool for the simultaneous interpretation of a variety of biological processes.

## Data Availability Statement

The datasets presented in this study can be found in online repositories. The names of the repository/repositories and accession number(s) can be found in the article/**Supplementary Material**.

## Ethics Statement

The animal study was reviewed and approved by the Animal Research Ethics Committee at Pukyong National University.

## Author Contributions

HR: Writing—Original draft, Methodology, Conceptualization, Formal analysis, Visualization, Investigation, Methodology. NK, YL, JP, and BK: Methodology, Formal analysis. ML and C-IP: Project administration, Methodology. D-HK: Writing—Review and Editing, Project administration, Funding acquisition, Conceptualization. All authors contributed to the article and approved the submitted version.

## Funding

Ministry of Oceans and Fisheries, Korea.

## Conflict of Interest

The authors declare that the research was conducted in the absence of any commercial or financial relationships that could be construed as a potential conflict of interest.
