# Supplementary material for: Dual-Organ Transcriptomic Analysis of Rainbow Trout Infected With Ichthyophthirius multifiliis Through Co-Expression and Machine Learning
Source: Front Immunol. 2021 Jul 8;12:677730. doi: 10.3389/fimmu.2021.677730 (PMC8296305; doi:10.3389/fimmu.2021.677730)
Supplement: Supplementary file 1 [file DataSheet_1.docx]

Supplementary Material

# Supplementary Figures


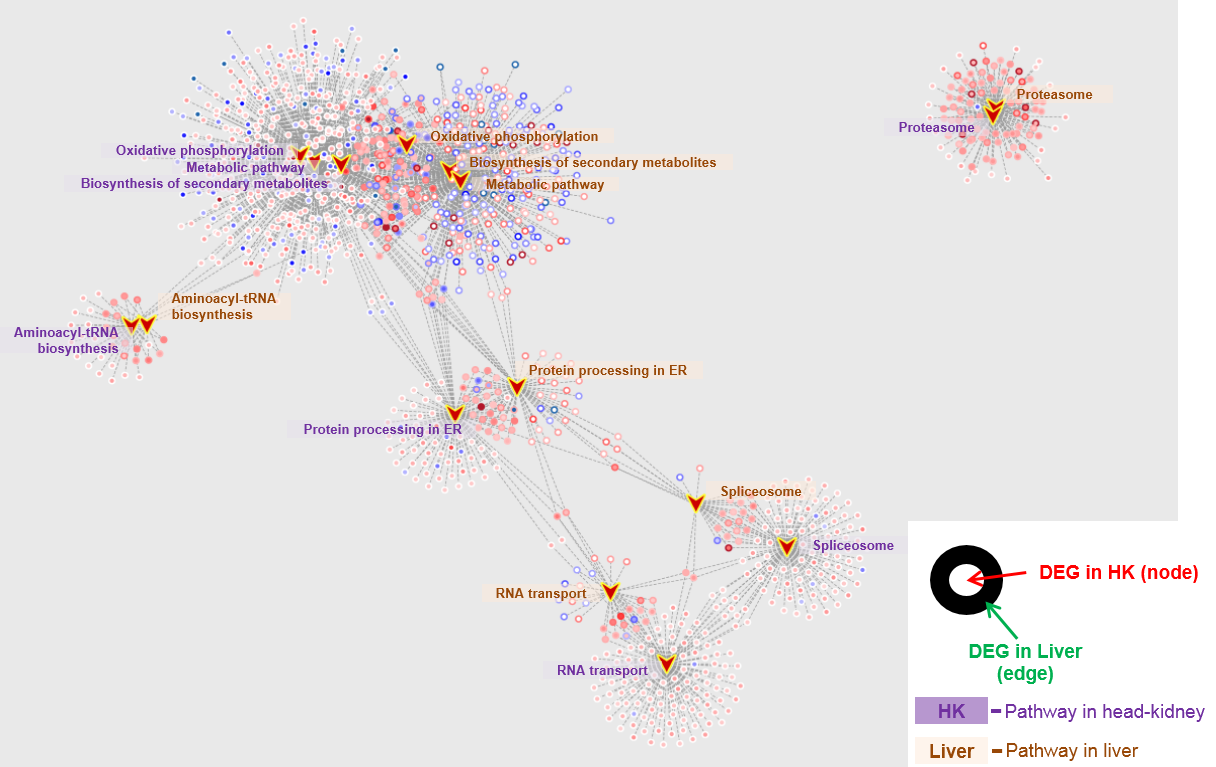


**Figure S1.** The primary pathways and relevant DEGs in PO-PO direction. The red check sign with yellow outline indicated pathway expressed in head-kidney (purple letters) and liver (brown letters). The red and blue color in node and edge were up- and down regulated DEGs.


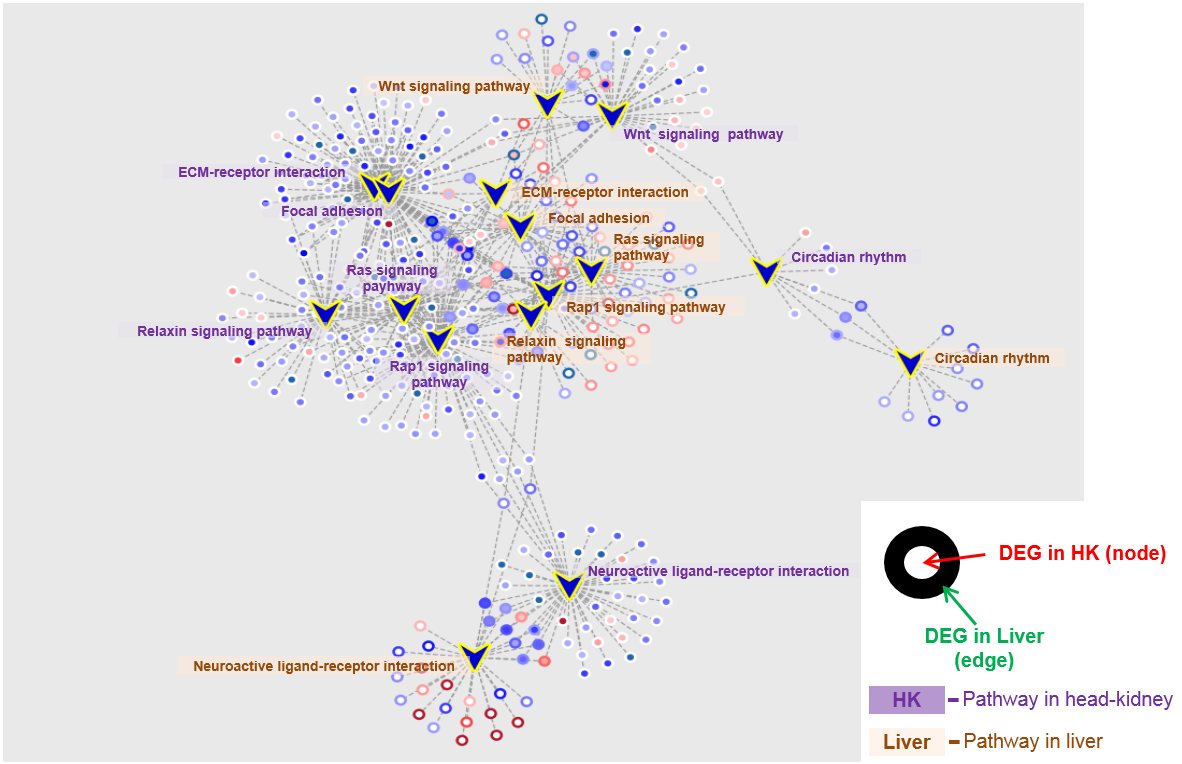


**Figure S2.** The primary pathways and relevant DEGs in NE-NE direction. The blue check sign with yellow outline indicated pathway expressed in head-kidney (purple letters) and liver (brown letters). The red and blue color in node and edge were up- and down regulated DEGs.


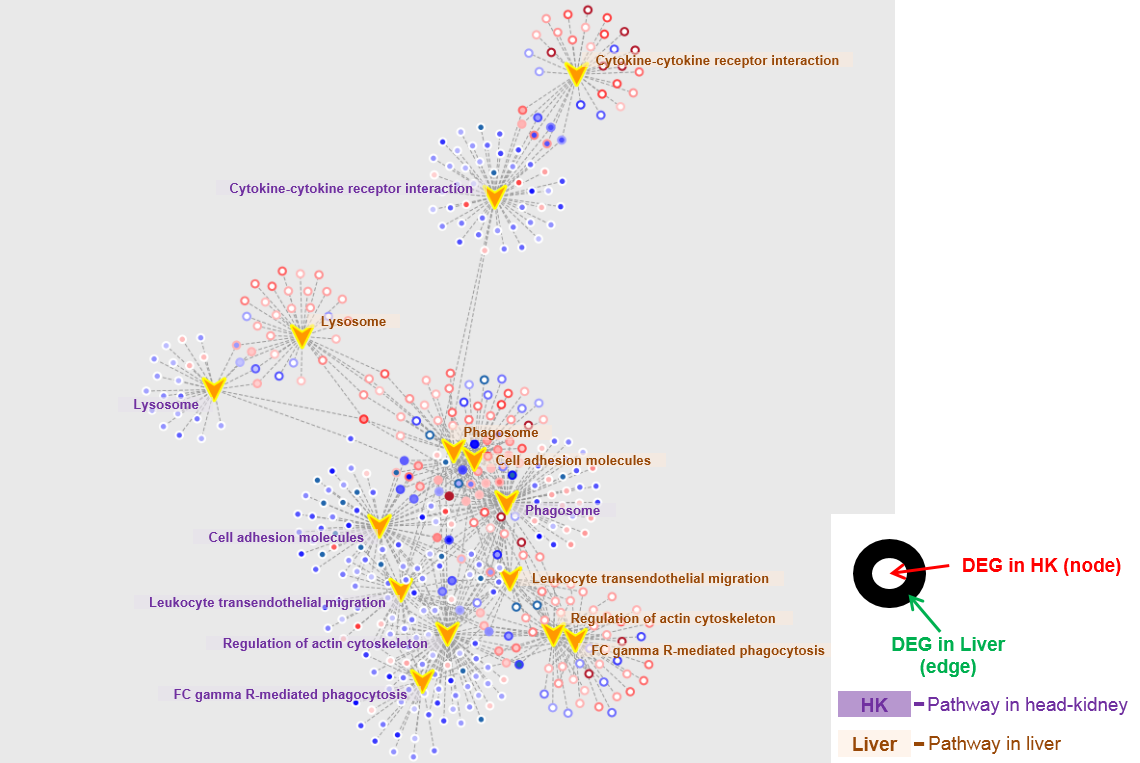


Figure S3. The primary pathways and relevant DEGs in NE-PO direction. The blue check sign with yellow outline indicated pathway expressed in head-kidney (purple letters) and liver (brown letters). The red and blue color in node and edge were up- and down regulated DEGs.


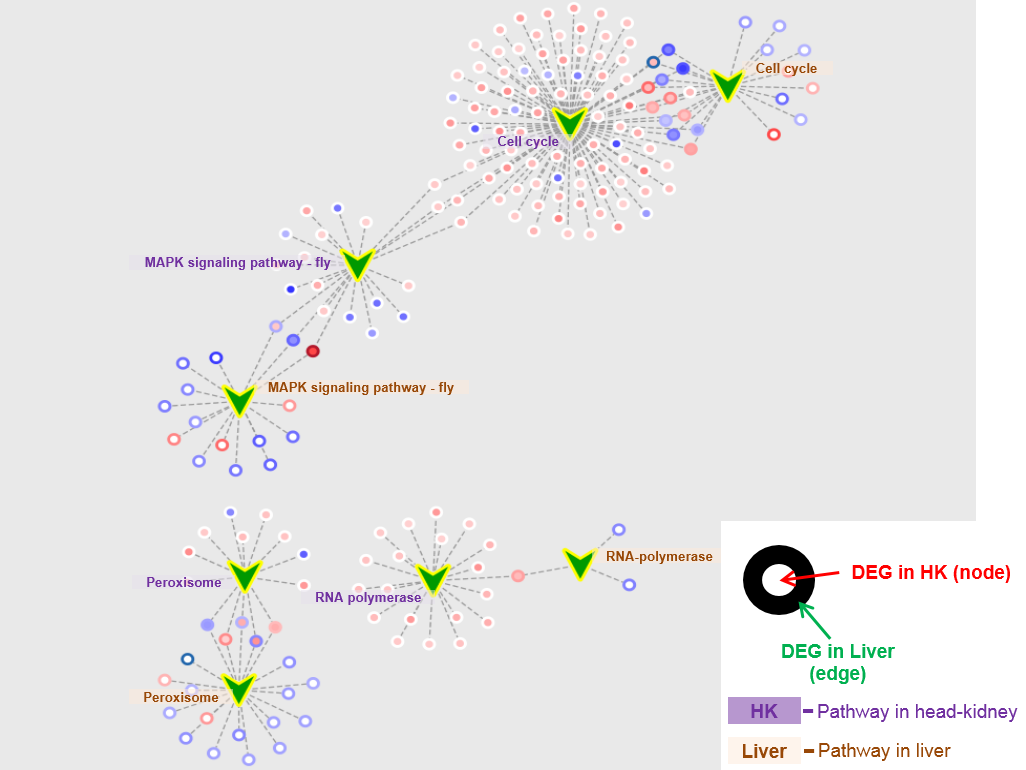


Figure S4. The primary pathways and relevant DEGs in PO-NE direction. The green check sign with yellow outline indicated pathway expressed in head-kidney (purple letters) and liver (brown letters). The red and blue color in node and edge were up- and down regulated DEGs.


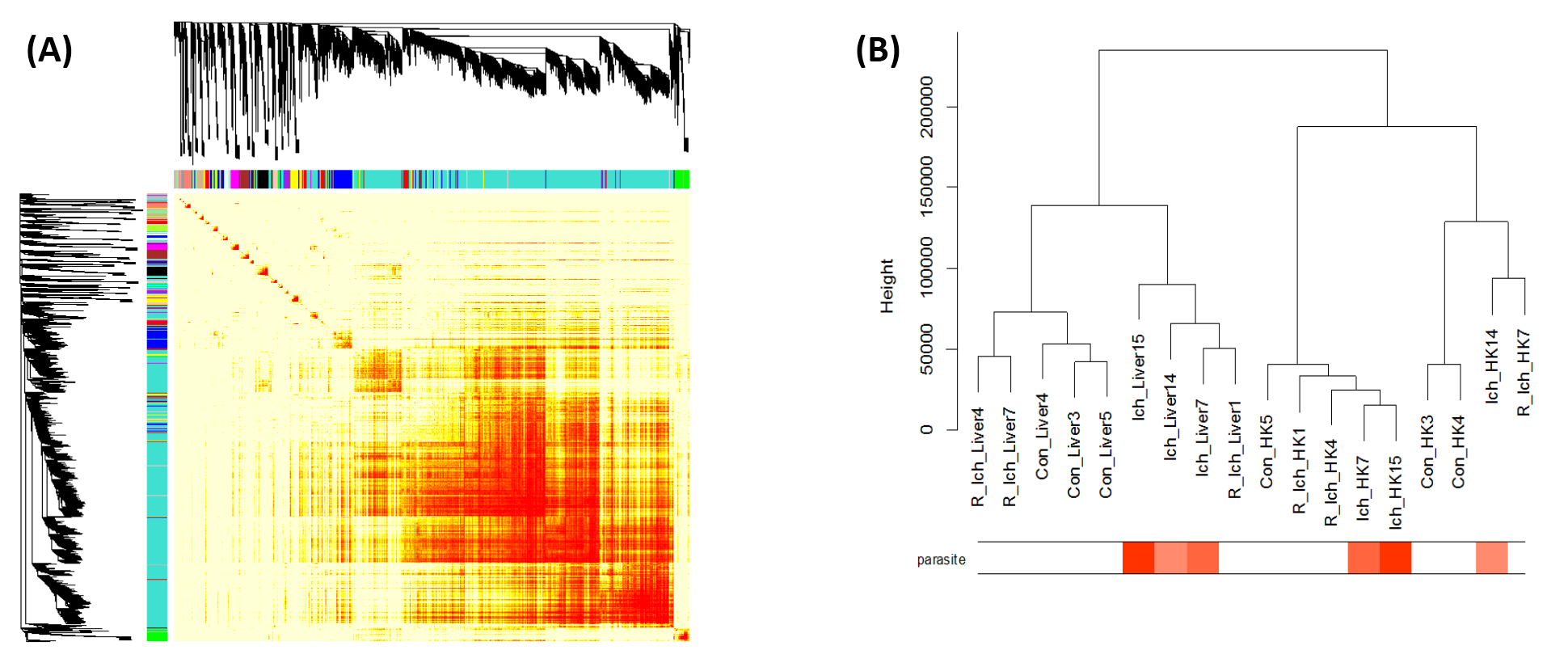


Figure S5. Network heat map and dendrogram of 19 different modules (A), and hierarchical clustering of 18 transcriptomics and degree of parasite infection (B).

# Supplementary Tables

**Table S1.** qPCR primer, probe and incubation condition used in this study.

| Gene | Primer, probe and incubation | Reference |
| --- | --- | --- |
| SAA | F: 5′- GGT GAA GCT GCT CAA GGT GCT AAA G -3′ | (42) |
|  | R: 5′- GCC ATT ACT GAT GAC TGT TGC TGC -3′ |  |
|  | 95 °C - 10min, (95 °C - 15s, 60 °C – 1min) x 40 cycles |  |
| Hap | F: 5′- TTA GGC ATC ACG CGA CAA TC -3′ | (40) |
|  | R: 5′- AGT CTG ATA CGT TCT GGA AGC C -3′ |  |
|  | 95 °C - 5min, (95 °C - 15s, 64 °C – 20s) x 40 cycles |  |
| HSP70 | F: 5′- AAC GGC ATT CTG AAC GTA GC -3′ | This study *  (GSONMG00064818001) |
|  | R: 5′- CTG TGC ATC ATC CTC AGC TTT G -3′ |  |
|  | 95 °C - 5min, (95 °C - 15s, 61 °C – 20s) x 40 cycles |  |
| Hmx | F: 5′- AAC GCA GCC TTT GTT TGT GC -3′ | (40) |
|  | R: 5′- TGG CAG ATA GGT CAA TGT CCA G -3′ |  |
|  | 95 °C - 5min, (95 °C - 15s, 64 °C – 20s) x 40 cycles |  |
| CD99 | F: 5′- GGG ACG AAA ACA CAA GAC TTC G -3′ | This study *  (GSONMG00029034001) |
|  | R: 5′- TCC TTT GCA GGT TCC TTT GG -3′ |  |
|  | 95 °C - 5min, (95 °C - 15s, 64 °C – 20s) x 40 cycles |  |
| IgT | F: 5′- AAC ATC ACC TGG CAC ATC AA -3′ | (42) |
|  | R: 5′- TTC AGG TTG CCC TTT GAT TC -3′ |  |
|  | 95 °C - 10min, (95 °C - 15s, 60 °C – 1min) x 40 cycles |  |
| Col | F: 5′- TGG TCA ATG TGA AGC AGC AG -3′ | This study *  (GSONMG00005028001) |
|  | R: 5′- TGC AGG AAA CGC TGG ATT TG -3′ |  |
|  | 95 °C - 5min, (95 °C - 15s, 64 °C – 20s) x 35 cycles |  |
| Ef-1α | F: 5′- GAT CCA GAA GGA GGT CAC CA -3′ | (42) |
|  | R: 5′- TTA CGT TCG ACC TTC CAT CC -3′ |  |
|  | 95 °C - 10min, (95 °C - 15s, 60 °C – 1min) x 40 cycles |  |
| Wap65-1 | F: 5′- ACA TGA GGG ACA CGA CCA TG -3′ | This study *  (GSONMG00038205001) |
|  | R: 5′- AAACCCTTGAACACATGGCC -3′ |  |
|  | Probe: /56-FAM/ TCA CAC GGG /ZEN/ TCT TTT GCT CGA CCG CTG CCA /3IABkFQ/ |  |
|  | 95 °C - 5min, (95 °C - 15s, 64 °C – 20s) x 40 cycles |  |

* The primers were designed in this study, and the gene accession number in the rainbow trout genome was listed in the reference column.

**Table S2.** The information of total reads and passed reads used in this study

| **Sample** | **Raw read** | **Clean read** | **Mapped read** | **Uniquely mapped** |
| --- | --- | --- | --- | --- |
| **Con3_HK** | 49,158,406 | 47,638,388 (96.9%) | 38,100,806 (80.0%) | 36,517,454 (76.7%) |
| **Con4_HK** | 48,209,518 | 46,726,828 (96.9%) | 37,018,647 (79.2%) | 35,367,773 (75.7%) |
| **Con5_HK** | 47,144,584 | 45,554,558 (96.6%) | 37,035,907 (81.3%) | 35,242,192 (77.4%) |
| **Con3_Liver** | 52,891,346 | 51,456,692 (97.3%) | 41,310,777 (80.3%) | 39,060,756 (75.9%) |
| **Con4_Liver** | 45,624,956 | 44,316,720 (97.1%) | 35,398,605 (79.9%) | 33,706,039 (76.1%) |
| **Con5_Liver** | 47,972,520 | 46,571,396 (97.1%) | 38,209,824 (82.0%) | 35,247,986 (75.7%) |
| **Ich7_HK** | 46,046,410 | 44,677,110 (97.0%) | 36,868,993 (82.5%) | 34,449,505 (77.1%) |
| **Ich14_HK** | 51,916,618 | 50,272,260 (96.8%) | 42,307,709 (84.2%) | 39,267,117 (78.1%) |
| **Ich15_HK** | 52,853,710 | 50,877,546 (96.3%) | 41,862,065 (82.3%) | 39,991,174 (78.6%) |
| **Ich7_Liver** | 46,774,606 | 45,601,510 (97.5%) | 35,903,858 (78.7%) | 33,766,003 (74.0%) |
| **Ich14_Liver** | 44,263,108 | 42,624,222 (96.3%) | 33,146,644 (77.8%) | 30,188,859 (70.8%) |
| **Ich15_Liver** | 46,603,834 | 45,418,080 (97.5%) | 37,185,720 (81.9%) | 34,647,612 (76.3%) |
| **R-Ich1_Hk** | 54,155,604 | 52,790,656 (97.5%) | 42,374,287 (80.3%) | 40,350,301 (76.4%) |
| **R-Ich4_Hk** | 52,555,496 | 51,250,874 (97.5%) | 41,460,537 (80.9%) | 39,551,917 (77.2%) |
| **R-Ich7_Hk** | 51,005,228 | 49,669,990 (97.4%) | 39,179,352 (78.9%) | 37,275,787 (75.0%) |
| **R-Ich1_Liver** | 50,637,822 | 49,269,814 (97.3%) | 40,782,795 (82.8%) | 38,830,256 (78.8%) |
| **R-Ich4_Liver** | 53,956,762 | 52,680,752 (97.6%) | 38,922,321 (73.9%) | 37,352,300 (70.9%) |
| **R-Ich7_Liver** | 48,098,548 | 46,761,502 (97.2%) | 36,806,939 (78.7%) | 35,316,071 (75.5%) |

**Table S3.** Pathway direction classification based on pathway expression tendency between Con vs Ich and Con vs R-Ich, and content ratio in head-kidney and liver.

| **Direction** | **Description** | **Head-kidney (Z-score)** | **Liver (Z-score)** |
| --- | --- | --- | --- |
| Up-regulation | Up-regulated KEGG pathways during *I. multifiliis* infection | 46% (163/357) | 51% (183/356) |
| Down-regulation | Down-regulated KEGG pathways during *I. multifiliis* infection | 51% (182/357) | 40% (144/356) |
| Etc. | Unchanged KEGG pathways during *I. multifiliis* infection | 3% (12/357) | 8% (29/356) |

**Table S4.** Top 10% pathway and Z-score for each stage direction in head-kidney during *I. multifillis* infection and recovery.

| **Direction** | **Pathway ID** | **Pathway** | **Head-kidney** | |
| --- | --- | --- | --- | --- |
|  |  |  | **Con vs Ich**  **(Z-score)** | **Con vs R-Ich**  **(Z-score)** |
| **Up-regulation** | ko01100 | Metabolic pathways | 9.5 | -1.2 |
|  | ko03013 | RNA transport | 8.6 | 1.0 |
|  | ko03040 | Spliceosome | 7.8 | -1.0 |
|  | ko01130 | Biosynthesis of antibiotics | 7.0 | 0.6 |
|  | ko00190 | Oxidative phosphorylation | 7.0 | 0.0 |
|  | ko01110 | Biosynthesis of secondary metabolites | 6.6 | 0.0 |
|  | ko03008 | Ribosome biogenesis in eukaryotes | 6.5 | 0.0 |
|  | ko03050 | Proteasome | 6.1 | 0.0 |
|  | ko04714 | Thermogenesis | 5.9 | 0.0 |
|  | ko04141 | Protein processing in endoplasmic reticulum | 5.8 | 1.0 |
|  | ko04111 | Cell cycle - yeast | 5.5 | 0.0 |
|  | ko03030 | DNA replication | 4.9 | 0.0 |
|  | ko04113 | Meiosis - yeast | 4.9 | 0.0 |
|  | ko03015 | mRNA surveillance pathway | 4.8 | 0.0 |
|  | ko01200 | Carbon metabolism | 4.7 | 1.0 |
|  | ko01120 | Microbial metabolism in diverse environments | 4.6 | 1.0 |
| **Down-regulation** | ko04510 | Focal adhesion | -5.6 | -1.7 |
|  | ko04015 | Rap1 signaling pathway | -5.5 | -1.7 |
|  | ko04512 | ECM-receptor interaction | -4.8 | -1.7 |
|  | ko04514 | Cell adhesion molecules (CAMs) | -4.3 | 0.0 |
|  | ko04611 | Platelet activation | -4.1 | -2.0 |
|  | ko04810 | Regulation of actin cytoskeleton | -4.0 | -1.4 |
|  | ko04060 | Cytokine-cytokine receptor interaction | -3.9 | -1.3 |
|  | ko04014 | Ras signaling pathway | -3.9 | 0.0 |
|  | ko04151 | PI3K-Akt signaling pathway | -3.7 | -1.7 |
|  | ko04916 | Melanogenesis | -3.6 | 0.0 |
|  | ko04927 | Cortisol synthesis and secretion | -3.4 | -1.7 |
|  | ko04670 | Leukocyte transendothelial migration | -3.4 | -1.4 |
|  | ko04660 | T cell receptor signaling pathway | -3.4 | 0.0 |
|  | ko04024 | cAMP signaling pathway | -3.2 | 0.0 |
|  | ko04022 | cGMP-PKG signaling pathway | -3.1 | 1.0 |
|  | ko04020 | Calcium signaling pathway | -3.1 | 0.6 |
|  | ko04540 | Gap junction | -3.0 | 0.0 |
|  | ko04725 | Cholinergic sy0pse | -3.0 | 0.0 |
|  | ko04261 | Adrenergic signaling in cardiomyocytes | -3.0 | 1.0 |

**Table S5.** Top 10% pathway and Z-score for each stage direction in liver during *I. multifillis* infection and recovery.

| **Direction** | **Pathway ID** | **Pathway** | **Liver** | |
| --- | --- | --- | --- | --- |
|  |  |  | **Con vs Ich**  **(Z-score)** | **Con vs R-Ich**  **(Z-score)** |
| **Up-regulation** | ko03050 | Proteasome | 6.1 | 2.2 |
|  | ko04141 | Protein processing in endoplasmic reticulum | 4.8 | 0.0 |
|  | ko04142 | Lysosome | 4.1 | -0.6 |
|  | ko00970 | Aminoacyl-tRNA biosynthesis | 3.5 | 0.0 |
|  | ko00190 | Oxidative phosphorylation | 3.4 | -3.0 |
|  | ko04666 | Fc gamma R-mediated phagocytosis | 3.1 | 1.0 |
|  | ko04144 | Endocytosis | 3.1 | 0.4 |
|  | ko04145 | Phagosome | 2.9 | 2.4 |
|  | ko04612 | Antigen processing and presentation | 2.7 | 2.0 |
|  | ko00510 | N-Glycan biosynthesis | 2.7 | 0.0 |
|  | ko04621 | NOD-like receptor signaling pathway | 2.6 | 1.6 |
|  | ko03060 | Protein export | 2.6 | 0.0 |
|  | ko04216 | Ferroptosis | 2.5 | 1.0 |
|  | ko00900 | Terpenoid backbone biosynthesis | 2.4 | -2.2 |
|  | ko04064 | NF-kappa B signaling pathway | 2.3 | 0.0 |
|  | ko01130 | Biosynthesis of antibiotics | 2.1 | -2.7 |
|  | ko01100 | Metabolic pathways | 1.9 | -3.4 |
|  | ko04650 | Natural killer cell mediated cytotoxicity | 1.8 | 2.0 |
| **Down-regulation** | ko01522 | Endocrine resistance | -3.1 | 0.8 |
|  | ko04013 | MAPK signaling pathway - fly | -2.7 | -0.4 |
|  | ko04710 | Circadian rhythm | -2.6 | 0.0 |
|  | ko04146 | Peroxisome | -2.5 | -1.0 |
|  | ko00310 | Lysine degradation | -2.3 | -1.4 |
|  | ko04080 | Neuroactive ligand-receptor interaction | -2.2 | 0.0 |
|  | ko04211 | Longevity regulating pathway | -1.9 | 0.0 |
|  | ko04919 | Thyroid hormone signaling pathway | -1.9 | 1.1 |
|  | ko04928 | Parathyroid hormone synthesis, secretion and action | -1.8 | 1.6 |
|  | ko04962 | Vasopressin-regulated water reabsorption | -1.7 | 0.0 |
|  | ko00280 | Valine, leucine and isoleucine degradation | -1.0 | -2.2 |
|  | ko04371 | Apelin signaling pathway | -0.9 | 2.2 |

**Table S6.** Top 10% pathway and Z-score for each co-expression direction in head-kidney and liver in Ich group.

| **Direction** | **Organ** | **Pathway ID** | **Pathway** | **Z-score (HK)** | **Z-score (Liver)** |
| --- | --- | --- | --- | --- | --- |
|  |  |  |  | **Con vs Ich** | **Con vs Ich** |
| **PO-PO** | **HK** | ko01100 | Metabolic pathways | 9.5 | 1.9 |
|  |  | ko03013 | RNA transport | 8.6 | 2.4 |
|  |  | ko03040 | Spliceosome | 7.8 | 3.4 |
|  |  | ko00190 | Oxidative phosphorylation | 7.0 | 3.4 |
|  |  | ko01110 | Biosynthesis of secondary metabolites | 6.6 | 1.5 |
|  | **Liver** | ko03050 | Proteasome | 6.1 | 6.1 |
|  |  | ko04141 | Protein processing in endoplasmic reticulum | 5.8 | 4.8 |
|  |  | ko00970 | Aminoacyl-tRNA biosynthesis | 4.4 | 3.5 |
|  |  | ko03040 | Spliceosome | 7.8 | 3.4 |
|  |  | ko00190 | Oxidative phosphorylation | 7.0 | 3.4 |
| **NE-NE** | **HK** | ko04510 | Focal adhesion | -5.6 | -2.3 |
|  |  | ko04015 | Rap1 signaling pathway | -5.5 | -1.1 |
|  |  | ko04512 | ECM-receptor interaction | -4.8 | -0.6 |
|  |  | ko04080 | Neuroactive ligand-receptor interaction | -4.0 | -2.2 |
|  |  | ko04014 | Ras signaling pathway | -3.9 | -1.1 |
|  | **Liver** | ko04710 | Circadian rhythm | -1.0 | -2.6 |
|  |  | ko04510 | Focal adhesion | -5.6 | -2.3 |
|  |  | ko04080 | Neuroactive ligand-receptor interaction | -4.0 | -2.2 |
|  |  | ko04310 | Wnt signaling pathway | -1.1 | -2.2 |
|  |  | ko04926 | Relaxin signaling pathway | -2.9 | -2.1 |
| **NE-PO** | **HK** | ko04514 | Cell adhesion molecules (CAMs) | -4.3 | 0.7 |
|  |  | ko04810 | Regulation of actin cytoskeleton | -4.0 | 1.5 |
|  |  | ko04060 | Cytokine-cytokine receptor interaction | -3.9 | 2.3 |
|  |  | ko04670 | Leukocyte transendothelial migration | -3.4 | 0.2 |
|  | **Liver** | ko04142 | Lysosome | -2.0 | 4.1 |
|  |  | ko04666 | Fc gamma R-mediated phagocytosis | -1.5 | 3.1 |
|  |  | ko04145 | Phagosome | -1.1 | 2.9 |
| **PO-NE** | **HK** | ko04110 | Cell cycle | 5.7 | -0.2 |
|  |  | ko03020 | RNA polymerase | 4.1 | -0.6 |
|  | **Liver** | ko04013 | MAPK signaling pathway - fly | 1.4 | -2.7 |
|  |  | ko04146 | Peroxisome | 1.9 | -2.5 |
| **Etc.** | **Only HK up** | ko04714 | Thermogenesis | 5.9 | 0.0 |
|  |  | ko03015 | mRNA surveillance pathway | 4.8 | 0.0 |
|  | **Only HK down** | ko04611 | Platelet activation | -4.1 | 0.0 |
|  |  | ko04151 | PI3K-Akt signaling pathway | -3.7 | 0.0 |
|  | **Only Liver up** | ko04614 | Renin-angiotensin system | 0.0 | 1.9 |
|  | **Only Liver down** | ko00790 | Folate biosynthesis | 0.0 | -1.5 |

**Table S7.** Top 10% pathway and Z-score for each co-expression direction in head-kidney and liver in R-Ich group.

| **Direction** | **Organ** | **Pathway ID** | **Pathway** | **Z-score**  **(HK)** | **Z-score (Liver)** |
| --- | --- | --- | --- | --- | --- |
|  |  |  |  | **Con vs R-Ich** | **Con vs R-Ich** |
| **PO-PO** | **HK** | ko04621 | NOD-like receptor signaling pathway | 2.6 | 1.6 |
|  |  | ko04612 | Antigen processing and presentation | 1.7 | 2.0 |
|  | **Liver** | ko04612 | Antigen processing and presentation | 1.7 | 2.0 |
|  |  | ko04020 | Calcium signaling pathway | 0.6 | 1.9 |
| **NE-NE** | **HK** | ko04512 | ECM-receptor interaction | -1.7 | -1.0 |
|  | **Liver** | ko01100 | Metabolic pathways | -1.2 | -3.4 |
| **NE-PO** | **HK** | ko00590 | Arachidonic acid metabolism | -2.0 | 1.3 |
|  |  | ko04611 | Platelet activation | -2.0 | 1.0 |
|  |  | ko04151 | PI3K-Akt signaling pathway | -1.7 | 0.6 |
|  | **Liver** | ko04371 | Apelin signaling pathway | -1.4 | 2.2 |
|  |  | ko04520 | Adherens junction | -1.0 | 2.2 |
|  |  | ko04015 | Rap1 signaling pathway | -1.7 | 1.9 |
|  |  | ko04670 | Leukocyte transendothelial migration | -1.4 | 1.9 |
| **PO-NE** | **HK** | ko04979 | Cholesterol metabolism | 1.7 | -0.9 |
|  | **Liver** | ko01130 | Biosynthesis of antibiotics | 0.6 | -2.7 |
|  |  | ko00280 | Valine, leucine and isoleucine degradation | 1.0 | -2.2 |
| **Etc.** | **Only HK up** | ko04141 | Protein processing in endoplasmic reticulum | 1.0 | 0.0 |
|  | **Only HK down** | ko04610 | Complement and coagulation cascades | -1.7 | 0.0 |
|  |  | ko04657 | IL-17 signaling pathway | -1.4 | 0.0 |
|  | **Only Liver up** | ko04145 | Phagosome | 0.0 | 2.4 |
|  |  | ko03050 | Proteasome | 0.0 | 2.2 |
|  |  | ko04514 | Cell adhesion molecules (CAMs) | 0.0 | 2.1 |
|  |  | ko04650 | Natural killer cell mediated cytotoxicity | 0.0 | 2.0 |
|  |  | ko04727 | GABAergic synapse | 0.0 | 2.0 |
|  | **Only Liver down** | ko01110 | Biosynthesis of secondary metabolites | 0.0 | -3.2 |
|  |  | ko00190 | Oxidative phosphorylation | 0.0 | -3.0 |
|  |  | ko04932 | Non-alcoholic fatty liver disease (NAFLD) | 0.0 | -2.3 |
|  |  | ko04714 | Thermogenesis | 0.0 | -2.0 |
|  |  | ko01212 | Fatty acid metabolism | 0.0 | -1.9 |

**Table S8.** Co-expression direction classification based on pathway expression tendency between head-kidney and liver, and content ratio in Con vs Ich and Con vs R-Ich comparison.

| **Direction** | **Description** | **Con vs Ich** | **Con vs R-Ich** |
| --- | --- | --- | --- |
| PO-PO | Pathways increased in both organs | 28% (104/372) | 14% (42/299) |
| NE-NE | Pathways decreased in both organs | 25% (93/372) | 7% (21/299) |
| NE-PO | Pathways decreased in head-kidney but increased in liver | 17% (65/372) | 23% (70/299) |
| PO-NE | Pathways increased in head-kidney but decreased in liver | 10% (38/372) | 8% (25/299) |
| Etc. | Pathways only increased in head-kidney | 6% (21/372) | 4% (13/299) |
|  | Pathways only decreased in head-kidney | 6% (24/372) | 8% (24/299) |
|  | Pathways only increased in liver | 4% (14/372) | 18% (53/299) |
|  | Pathways only decreased in liver | 3% (13/372) | 17% (51/299) |
